# Indirect comparisons of ranibizumab and dexamethasone in macular oedema secondary to retinal vein occlusion

**DOI:** 10.1186/1471-2288-14-140

**Published:** 2014-12-22

**Authors:** Howard HZ Thom, Gorana Capkun, Richard M Nixon, Alberto Ferreira

**Affiliations:** School of Social and Community Medicine, University of Bristol, Bristol, UK; Novartis Pharma, Basel, Switzerland

**Keywords:** Ranibizumab, Dexamethasone, Retinal vein occlusion, Central retinal vein occlusion, Branch retinal vein occlusion, Macular oedema, Evidence synthesis, Indirect comparison

## Abstract

**Background:**

Two treatments, ranibizumab and dexamethasone implant, for visual impairment due to macular oedema (ME) secondary to retinal vein occlusion (RVO) have recently been studied in clinical trials. There have been no head to head comparisons of the two treatments, and improvement measured as gain in Best Corrected Visual Acuity (BCVA) was reported using different outcomes thresholds between trials. To overcome these limitations, and inform an economic model, we developed a combination of a multinomial model and an indirect Bayesian comparison model for multinomial outcomes.

**Methods:**

Outcomes of change from baseline in BCVA for dexamethasone compatible with those available for ranibizumab, reported by 4 randomised controlled trials, were estimated by fitting a multinomial distribution model to the probability of a patient achieving outcomes in a range of changes from baseline in BCVA (numbers of letters) at month 1. A Bayesian indirect comparison multinomial model was then developed to compare treatments in the Branch RVO (BRVO) and Central RVO (CRVO) populations.

**Results:**

The multinomial model had excellent fit to the observed results. With the Bayesian indirect comparison, the probabilities of achieving ≥20 letters, with 95% credible intervals, at month 1 in patients with BRVO were 0.191 (0.130, 0.261) with ranibizumab and 0.093 (0.027, 0.213) with dexamethasone. In patients with CRVO, probabilities were 0.133 (0.082, 0.195) (ranibizumab) and 0.063 (0.016, 0.153) (dexamethasone). Probabilities of a gain in ≥10 letters in BRVO patients were 0.500 (0.365, 0.650) v 0.459 (0.248, 0.724) and in CRVO patients 0.459 (0.332, 0.602) v 0.498 (0.263, 0.791) for ranibizumab and dexamethasone treatments respectively. The comparisons also favoured ranibizumab at month 6 although changes to therapies after month 3 may have introduced bias.

**Conclusion:**

The newly developed combination of multinomial and indirect Bayesian comparison models indicated a trend for ranibizumab association with a greater percentage of ME patients achieving visual gains than dexamethasone at months 1 and 6 in a common clinical context, although results were not classically significant. The method was a useful tool for comparisons of probability distributions between clinical trials that reported events on different categorical scales and estimates can be used to inform economic models.

## Background

Macular oedema secondary to retinal vein occlusion (RVO) is the second most frequent major retinal vascular disease after diabetic retinopathy and is also one of the most common causes of sudden visual loss [1, 2]. Branch retinal vein occlusion (BRVO) involving only a single retinal vein is the most common [3], while central retinal vein occlusion (CRVO) is less common but is more serious and carries a high risk of complications and vision loss [4]. Until recently, only grid laser photocoagulation was available to treat macular oedema secondary to BRVO while there were no effective treatments for macular oedema secondary to CRVO [5, 6]. Recently, the introduction of ranibizumab and of dexamethasone implant has widened the therapeutic choice. Both treatments have been shown to be effective against the visual acuity loss that is associated with both diseases, ranibizumab in the BRAVO [7] and CRUISE [8] trials in BRVO and CRVO, respectively, and dexamethasone implant in the GENEVA trials, two identically designed trials in BRVO and CRVO, from which the results were pooled and published together [9–11].

We were interested in building a health economic model to compare the cost-effectiveness of ranibizumab compared with dexamethasone to better inform healthcare decision making in bodies such as the National Institute of Health and Care Excellence in England and Wales and the Pharmaceutical Benefits Advisory Committee in Australia. This model would use change in best corrected visual acuity (BCVA), measured in letters and categorised into multiples of 10, at 1 month as its primary outcome. We chose 10 letters change in BCVA as recent research has shown that decrements of this magnitude were significantly associated with changes in quality of life [12]. However, no data are available from a direct comparison between the two therapies in either BRVO or CRVO so it is difficult estimate appropriate transition probabilities. The results from the trials on BRVO and CRVO are not directly comparable: although the trials measured change in BCVA, the primary end point in the ranibizumab trials was change from baseline after six months, whereas the GENEVA trials used time to ≥15 points improvement in BCVA. Hence, to assess the effectiveness of ranibizumab compared with dexamethasone implant would appear to require a head-to-head clinical trial, which is associated with significant use of time and resources.

To overcome these difficulties and inform our economic model, we developed a method for comparing the available results from the clinical trials with ranibizumab and dexamethasone implant. A two-step process was designed. The first step was to estimate the outcomes of change in BCVA for dexamethasone that were compatible with those available for ranibizumab using a multinomial model. In the second step, a Bayesian indirect comparison of dexamethasone against ranibizumab was performed, using multinomial probabilities estimates from step 1.

## Methods

The objective of the developed model was to allow for efficacy comparisons between ranibizumab (0.5 mg) and dexamethasone (0.7 mg) implant based on data from separate randomised studies. The doses were chosen based on current market availability of the respective treatments and the doses used in the clinical trials. The primary end point for the comparison was the percentages of patients reaching different levels of change from baseline in BCVA at month 1. The choice of primary endpoint, multiples of 10 letters change in BCVA at 1 month, was driven by the need to inform transition probabilities in our health economic model with a cycle length of 1 month. The model we developed could have been used to compare treatments on other increments, such as the 5 or 15 reported by GENEVA, but these would not have been useful for the health economic model. The 10 letters change BCVA endpoints were reported in the ranibizumab trials, and month 1 was selected as this was the earliest time point at which response was measured in all trials. In addition, patients in the BRAVO trial of ranibizumab were offered laser photocoagulation treatment after month 3, which might introduce bias in favour of ranibizumab into analyses from this time point onwards.

### Available data

Novartis Pharma and the York Health Economics Consortium shared the results of a systematic literature search performed on 18 November 2010 in core medical databases (Medline, EMBASE, the Cochrane Library, Cumulative Index to Nursing and Allied Health Literature [CINAHL]) and relevant websites including the International Clinical Trials Registry Platform (ICTRP) and the Association of Research and Vision and Ophthalmology (ARVO). The following Patients Interventions Comparators Outcomes Studies (PICOS) criteria were followed for this search

- **P:** Patients with clinically significant BRVO or CRVO.
- **I:** Ranibizumab or Dexamethasone IVT
- **C:** Any of the above plus supportive care, grid pattern photocoagulation, sham injections, or mixed treatment comparisons
- **O:** At least one of “Mean change in BCVA from baseline” and “Number of patients gaining ≥ 10 letters from baseline to 6 months”.
- **S:** Randomised controlled trials

Only studies published in English were included. This search identified three trials of ranibizumab, BRAVO in BRVO [7], and ROCC [13] and CRUISE [8] in CRVO, and the two GENEVA trials of dexamethasone in BRVO and CRVO [9–11]. The quality and potential risk of bias of included studies was assessed according to the minimum criteria specified by the NICE guidelines [14]. Key points assessed included method of randomization, blinding protocols and baseline patient demographics.

We based our analysis on the BRAVO, CRUISE and GENEVA trials. We could not include ROCC in our indirect comparison as it reported only mean and standard deviation of BCVA change and not percentages of patients achieving different categories of change. ROCC included only 29 patients while BRAVO, CRUISE and GENEVA each included ≥ 260 patients so there is not a great loss of evidence. The included trials were prospective randomised, multicentre, masked, and sham-controlled, with the primary end points evaluated at 6 months. All trials further reported mean change with 95% confidence intervals (CI) in BCVA from baseline at months 1 and 3 for the actively and sham-treated populations. Results in this paper concentrate on our primary endpoint of 1 month, which was the first available in the trials and required by a health economic model, and the latest end point of 6 months, which was the primary endpoint of the included trials.

In GENEVA, 291 BRVO and 136 CRVO patients, respectively, received dexamethasone 0.7 mg and 279 BRVO and 147 CRVO patients, respectively, received sham. Although the BRVO and CRVO populations were pooled for the published analysis, we used the GENEVA data as if it were two separate trials. BRAVO included 131 patients receiving ranibizumab and 132 receiving sham injections; in CRUISE 130 patients received ranibizumab and 130 sham injections, respectively.

**Figure 1:**
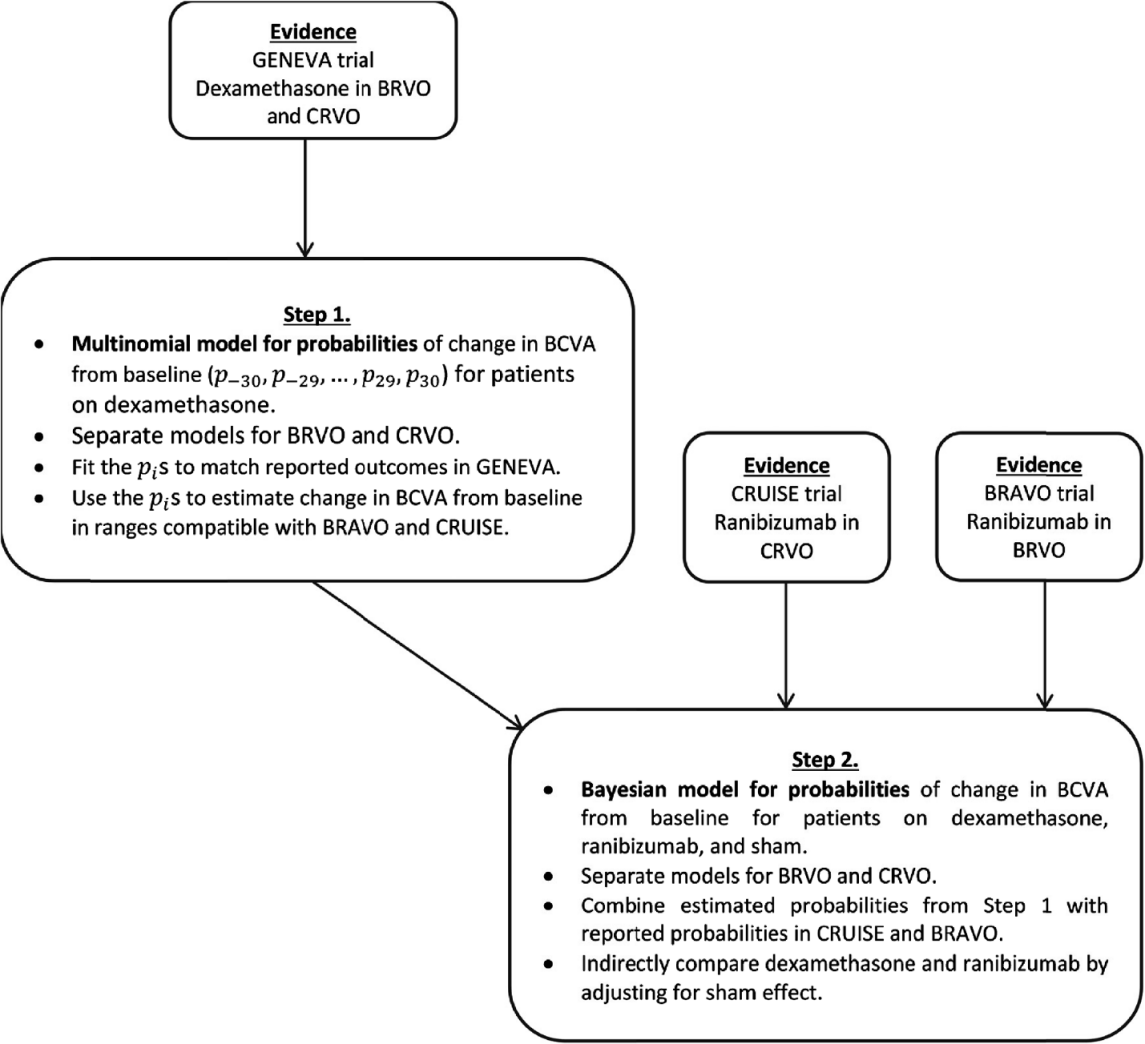
**Diagram of 2-step indirect comparison methodology with evidence sources based on multinomial model for probabilities of change in BCVA from baseline for dexamethasone and Bayesian indirect comparison of dexamethasone with ranibizumab.**

The changes in BCVA from baseline to month 1 for ranibizumab from the BRAVO and CRUISE trials are summarized in Table 1 and published results from the GENEVA trials for dexamethasone in Table 2. For the analysis, changes in BCVA at month 1 were stratified into 5 groups in increments of 10 letters; the classes of response used in the ranibizumab trials (Table 1). These thresholds were selected because gains in 10 letters are considered clinically relevant in this population [12], and to maintain symmetry with respect to the no-gain category. The indirect comparison was performed in two steps (Figure 1).

**Table 1:** **Reported proportions of categorical change from baseline at month 1 in BCVA in patients with BRVO and CRVO for patients on ranibizumab and sham**

	BRVO			CRVO		
Improvement or worsening at month 1	Ranibizumab (n1 = 131)	Sham (n2 = 132)	Relative risk (95% CI) ^‡^	Ranibizumab (n1 = 130)	Sham (n2 = 130)	Relative risk (95% CI) ^‡^
Gain ≥ 20 letters	0.191	0.038	5.04 (1.99, 12.76)	0.131	0.031	4.25 (1.47, 12.29)
Gain ≥ 10 letters and <20 letters	0.313	0.197	1.59 (1.04, 2.44)	0.331	0.100	3.31 (1.87, 5.85)
Loss <10 letters and gain <10 letters	0.489	0.705	0.69 (0.56, 0.85)	0.523	0.762	0.69 (0.57, 0.83)
Loss ≥10 letters and <20 letters	0.008	0.045	0.17 (0.02, 1.38)	0.015	0.077	0.20 (0.04, 0.90)
Loss ≥20 letters	0.000	0.015	0.00 (0.00, 0.00)	0.000	0.031	0.00 (0.00, 0.00)

^‡^Confidence intervals for relative risk calculated via delta method and reported patient numbers.

**Table 2:** **Reported proportions of categorical change from baseline at month 1 in BCVA in patients with BRVO and CRVO on dexamethasone and sham**

	BRVO			CRVO		
Improvement or worsening at month 1	Dexamethasone (n1 = 291)	Sham (n2 = 279)	Relative risk* (95% CI) ^‡^	Dexamethasone (n1 = 136)	Sham (n2 = 147)	Relative risk* (95% CI) ^‡^
Gain ≥15 letters	0.213	0.079	2.70 (1.71, 4.27)	0.213	0.068	3.13 (1.59, 6.19)
Gain ≥5 and <15 letters	0.474	0.369	1.28 (1.06, 1.56)	0.412	0.245	1.68 (1.19, 2.38)
Loss <5 letters and gain <5 letters	0.258	0.441	0.58 (0.46, 0.74)	0.301	0.483	0.62 (0.46, 0.85)
Loss ≥5 letters and <15 letters	0.055	0.097	0.57 (0.31, 1.03)	0.037	0.136	0.27 (0.10, 0.70)
Loss ≥15 letters	0.000	0.014	0.000 (0.00, 0.00)	0.037	0.068	0.54 (0.19, 1.54)
Gain ≥10 letters	0.426	0.201	2.12 (1.62, 2.78)	0.456	0.122	3.74 (2.33, 5.96)
Mean improvement (letters)^¶^	8.5	3.8		7.2	0.4	
SD improvement§	7.916	7.916		10.721	10.721	

Source: GENEVA, 2010; NICE manufacturer submission [10].

*Ratio of treatment (dexamethasone) probability to control (Sham) probability. § Estimated from reported limits on change BCVA.

^¶^Mean improvements were reported up to only 1 decimal place.

^‡^Confidence intervals for relative risk calculated via delta method and reported patient numbers.

Selected baseline characteristics from the treatment and sham arms of GENEVA, BRAVO and CRUISE are reported in Table 3. There were a limited number of common baseline characteristics reported across the trials but those that were reported, such as age, race, gender, and baseline BCVA, appeared to be similar and have comfortably overlapping ranges. Note that the definition of macular oedema differed across the studies so this characteristic is not strictly comparable.

**Table 3:** **Selected baseline characteristics reported by the included studies**

Baseline characteristic	GENEVA		BRAVO		CRUISE	
	Sham (n = 426)	Dexamethasone (0.7 mg) (n = 427)	Sham (n = 132)	Ranibizumab (0.5 mg) (n = 131)	Sham (n = 130)	Ranibizumab (0.5 mg) (n = 130)
Mean age (range) (yrs)	63.9 (31–91)	64.7 (33–90)	65.2 (26–89)	67.5 (41–91)	65.4 (20–91)	67.6 (40–91)
Male (%)	56.3	50.8	56.1	54.2	55.4	61.5
Race Caucasian (%)	74.6	75.2	81.8	81.7	86.9	83.1
Mean duration macular oedema* (range)	156.1 (19–374)	157.6 (19–374)	114.7 (0–496)	102.3 (0–403)	89.9 (0–434))	102.3 (0–837)
Mean baseline visual acuitity BCVA (sd)	54.8 (9.86)	54.3 (9.93)	54.7 (12.2)	53.0 (12.5)	49.2 (14.7)	48.1 (14.6)
Mean baseline retinal thickness (SD) (µm)	539 (186)	562 (188)	NR	NR	NR	NR
Fellow eye BCVA (ETDRS letters), mean (SD)	NR	NR	79.8 (17.4)	81.4 (13.8)	78.9 (18.6)	78.8 (17.4)

*In BRAVO and CRUISE studies, this was defined as “Months from RVO diagnosis to screening”. For simplicity of comparison, months are multiplied by 31 to convert to days.

### Step 1. Multinomial model for dexamethasone

The published GENEVA results include only a selection of summary statistics of improvement in BCVA from baseline to month 1 (Table 2). The first step was therefore to estimate outcomes for dexamethasone on a scale compatible with that for ranibizumab. This was done by fitting a multinomial distribution to the probability of a patient achieving the 5 different outcomes used in the ranibizumab trials. A multinomial distribution was chosen, as opposed to a gamma or beta distribution, as it provides maximum flexibility for the data available.

Relative risks and mean changes from baseline in BCVA at month 1 were used to fit separate multinomial distributions for dexamethasone and sham, separately for BRVO and CRVO, assuming a non-zero probability for each integer change in baseline from −30 letters (a loss) to +30 letters (a gain).

The standard deviations for changes from baseline in each group were estimated using the approach shown in the Appendix. The estimated standard deviation was s = 7.916 for BRVO and s = 10.721 for CRVO.

Labelling the probabilities (for dexamethasone or its sham) as *p*_−â€‰30_,â€‰*p*_−â€‰29_,â€‰â€¦,â€‰*p*_29_,â€‰*p*_30_, we can calculate the mean of the distribution as:


and its standard deviation as:


Probabilities of being in a particular range of change in letters from baseline are defined to be simple sums of the *pi* s (eg.  for the range ≥5 and <15 letters gain).

The set of 9 ranges below represent the greatest common factor of ranges between those for ranibizumab (Table 1) and those for the dexamethasone implant and its sham (Table 2). They correspond to probabilities *qj* and were used to fit the multinomial model.


Assuming the *pi* are uniform within the ranges (eg. *p*_1_ = *q*_5_/10), the means and standard deviations from the distribution of *qj* s can be calculated. The outcomes on which we have data, such as loss ≥5 and <15 letters, are at multiples of the granularity of the above model. For this reason, the assumption of uniformity within the ranges makes no difference to model fit as the same *qi* would be found without this assumption.

Using Microsoft Excel and the â€˜solver’ add-in, choosing the BRG algorithm for nonlinear optimization [15], with differences between consecutive iterations less than 0.0001 as the convergence criterion, the multinomial distributions for dexamethasone and its sham for BRVO and CRVO patients were fitted by minimizing the root mean squared error (RMSE) between the model output and data on the means, standard deviations, 6 probabilities reported in Table 2, and the corresponding relative risks of dexamethasone versus sham, subject to the following constraints:


All *qi* ≥ 0

These constraints ensure the *qi* form a multinomial probability distribution. Multiplying these probabilities *qi* by the total number of patients gives estimated counts of patients achieving each change in BCVA, which are used in Step 2.

### Step 2. Indirect comparison of ranibizumab against dexamethasone

Step 2 comprised the derivation of relative risks for ranibizumab and dexamethasone and the indirect comparison of dexamethasone against ranibizumab using the probability estimates for dexamethasone from Step 1. This requires the synthesis of evidence on multinomial outcomes in a competing risk situation. Several methods have been proposed for this problem [16], including a method based on normal approximations of a dichotomous outcome for pairwise meta-analysis [17] and a more general approach for multinomial outcomes [16, 18]. This latter method could be applied in for a similar situation case but as it works with log hazards and log hazard ratios, it would not provide estimation of probabilities and relative risks that are required by the health economic model in addition of being easier to interpret; the same statistics were reported in BRAVO, CRUISE and GENEVA.

To derivate relative risks for ranibizumab and dexamethasone, we denoted the relative risk of ranibizumab (Treatment) versus its sham (Control) as


where  and  are the probabilities of achieving a certain outcome at month 1 of treatment in the ranibizumab or sham arms of the BRAVO or CRUISE trials, and the relative risk of dexamethasone versus its sham as


where  and  are the probabilities of achieving a certain outcome at month 1of treatment in the dexamethasone or sham arms of the GENEVA trial.

Using the standard Bucher [19] method to calculate the indirect relative risk, adjusting for the sham treatment, of dexamethasone versus ranibizumab would lead to indirect probabilities of dexamethasone patients achieving selected outcomes in the setting of the CRUISE trial of >1. In addition, the Bucher method would treat each probability independently resulting in the sum of all probabilities being >1 which is inconsistent with the multinomial nature of the data. The method of multiplicatively rescaling the probabilities (i.e. dividing all probabilities by their total) would lead to serious distortions of the results, so was not used. In addition, these simple techniques tend to suffer from divide by zero errors. To overcome this, a Bayesian approach for multinomial outcomes was developed as described below.

Counts of patients in the five different categories of BCVA gain or loss were modelled by a multinomial distribution:


Where *i* indicates the trial with *i* = 1 for BRAVO or CRUISE in BRVO and CRVO, respectively, and *i* = 2 for GENEVA in both BRVO and CRVO.  and  are the number of patients in the control and treatment populations.  and  are vectors of five patient counts in each outcome category for the control and treatment populations of the *i*^th^ trial. For ranibizumab, these were calculated by multiplying observed probabilities from BRAVO and CRUISE by the corresponding patient counts  and , while for dexamethasone we multiplied the GENEVA patient counts by the estimated probabilities from Step 1.  and  are the vectors of probabilities of being in the different categories in the control and treatment populations which were estimated by our Bayesian model. Zero counts are not an issue in this model as our inference is on the probabilities, which are never exactly zero due to prior assumptions.

A Dirichlet (1,1,1,1,1) prior was placed on all control probabilities  and on the treatment probabilities  for ranibizumab. This constrained all outcome probabilities to sum to 1, to be <1 and to be non-zero.  for dexamethasone are specified by their relation to the indirect probabilities, , which are the probabilities we would expect to observe for patients treated with dexamethasone in the ranibizumab trial populations. This use of indirect probabilities accounts for differences in expected responses to common treatments across trial populations, providing a common clinical context for comparison of dexamethasone with ranibizumab.

For *i* = 1,2 the control and treatment probabilities are related via the relative risks:


where *j* = 1,â€¦,5 for five categories of BCVA change from baseline.

This is sufficient for *i* = 1 (ranibizumab) as a prior was placed on , but requires further specification for dexamethasone (*i* =2). The relative risk for dexamethasone is defined as


which is the ratio of the indirect probability of dexamethasone in the ranibizumab population to the probability of the control in the ranibizumab population. As only the dexamethasone relative risks are assumed common across trial populations, and not the absolute probabilities, this method preserves randomization. Dirichlet (1,1,1,1,1) priors are placed on the indirect probabilities  constraining them to sum to 1, to be <1, and to be non-zero. In this way, the indirect comparison is adjusting for the sham treatment in each study in a similar way to the Bucher method. As no prior is placed on the vector of probabilities in the dexamethasone population, they are explicitly constrained to sum to 1 via the rescaling:


This model was fit in the WinBUGS software package [20], with results sampled from 100 000 simulations following a burn-in of 50 000. Posterior means and 95% credible intervals (Bayesian confidence intervals) for the parameters were obtained.

## Results

The estimated probabilities, means and standard deviations of change from baseline in BCVA at 1 month (30 days) for patients with BRVO and CRVO on dexamethasone based on the multinomial model are summarized in Table 4. Iterative numerical minimization of the RMSE was stopped when chains were judged to have converged, based on the condition that differences between consecutive iterations were less than 0.0001. The total RMSE of the model, based on difference between estimated and observed probabilities, relative risks, means and standard deviations, was 0.323 for BRVO and 0.113 for CRVO. These were almost entirely driven by differences between the probabilities and risk ratios for dexamethasone in BRVO and the means and standard deviations in CRVO. Fit of this model can be further judged by comparing the model outputs of Table 4 to those reported from the GENEVA trial in Table 2. The model had excellent fit to the observed results.

**Table 4:** **Estimated probabilities, means and standard deviations of the change in number of letters from baseline at month 1 for dexamethasone and sham for BRVO and CRVO for ranges compatible with GENEVA**

	BRVO			CRVO		
Improvement or worsening at month 1	Dexamethasone	Sham	Relative risk*	Dexamethasone	Sham	Relative risk*
Gain ≥15 letters	0.231	0.086	2.70	0.213	0.068	3.13
Gain ≥5 and <15 letters	0.488	0.373	1.31	0.412	0.245	1.68
Loss <5 letters and gain <5 letters	0.257	0.440	0.58	0.301	0.482	0.62
Loss ≥5 letters and <15 letters	0.024	0.095	0.25	0.037	0.135	0.27
Loss ≥15 letters	0.000	0.006	0	0.037	0.068	0.54
Gain ≥10 letters	0.434	0.202	2.15	0.456	0.122	3.74
Mean improvement (letters)	8.498	3.799		7.200	0.400	
SD improvement^§^	7.917	7.916		10.721	10.721	

*Ratio of treatment (dexamethasone) probability to control (Sham) probability. ^§^Estimated from reported limits on change BCVA.

**Figure 2:**
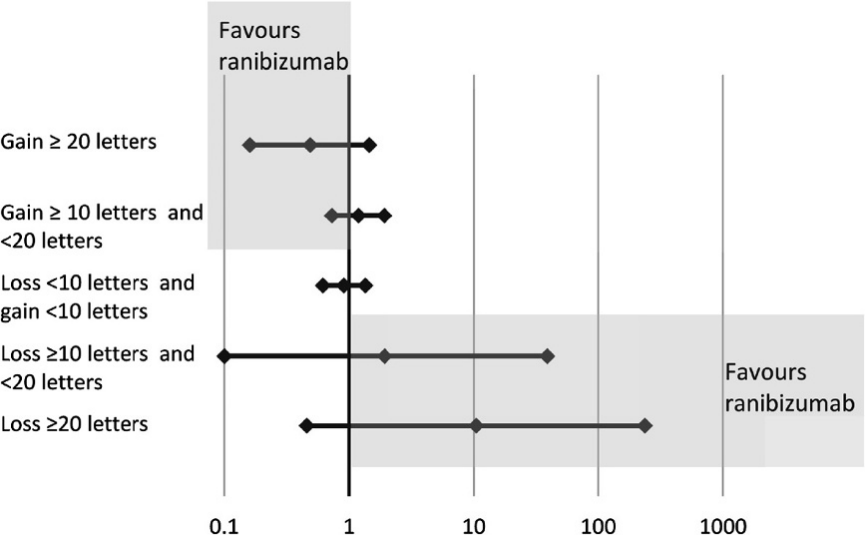
**Forest plot of indirect relative risk ±95% CI at Month 1 for patients with BRVO.** In the upper point estimates, values <1 would indicate greater chance of improving with ranibizumab; in the bottom point estimates, values >1 would indicate greater risk of worsening with dexamethasone.

**Figure 3:**
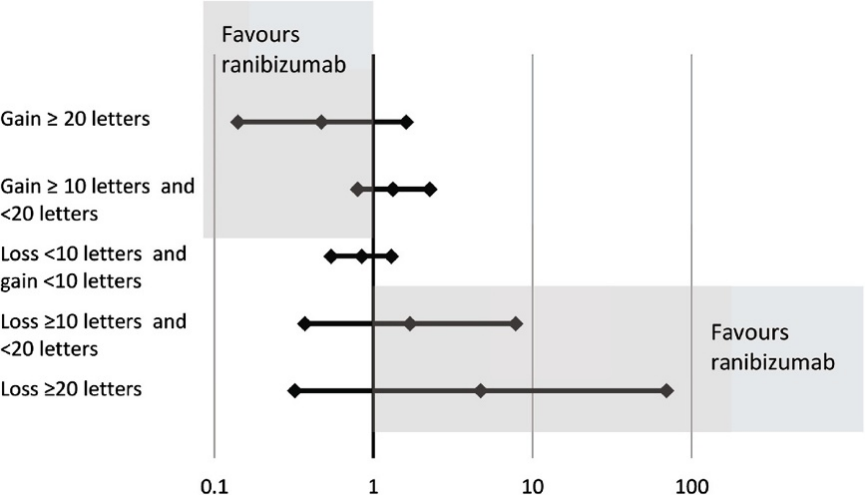
**Forest plot of indirect relative risk ±95% CI at Month 1 for patients with CRVO.** In the upper point estimates, values <1 would indicate greater chance of improving with ranibizumab; in the bottom point estimates, values >1 would indicate greater risk of worsening with dexamethasone.

Results of the Bayesian indirect comparison are reported in Tables 5 and 6 for BRVO and CRVO, respectively. Additionally, forest plots of indirect probabilities are presented in Figures 2 and 3 for BRVO and CRVO, respectively. The model showed overall greater response to ranibizumab than to dexamethasone: the estimated probability of achieving ≥20 letters improvement from baseline in BCVA at month 1 was 0.191 in BRVO patients on ranibizumab and 0.093 on dexamethasone. For CRVO patients the probabilities were 0.133 with ranibizumab and 0.063 with dexamethasone, respectively. Differences between treatments were more pronounced for the greatest responses: the estimated probabilities of ≥10 letters improvement from baseline in BCVA at month 1 for BRVO patients were 0.500 on ranibizumab v 0.459 on dexamethasone and in CRVO patients 0.459 on ranibizumab v 0.498 on dexamethasone.

**Table 5:** **Bayesian method estimates of indirect probabilities of passing different visual acuity thresholds at month 1 for patients with BRVO**

Improvement or worsening at month 1	Probability for Group ^§^ (95% CrI)		Relative risk* (95% CrI)
	Ranibizumab	Dexamethasone	
Gain ≥20 letters	0.191 (0.130, 0.261)	0.093 (0.027,0.213)	0.49 (0.16, 1.45)
Gain ≥10 letters and <20 letters	0.309 (0.235,0.389)	0.366 (0.221, 0.511)	1.19 (0.73, 1.92)
Loss <10 letters and gain <10 letters	0.478 (0.395, 0.561)	0.436 (0.282, 0.579)	0.91 (0.62, 1.35)
Loss ≥10 letters and <20 letters	0.015 (0.002,0.041)	0.028 (0.000, 0.133)	1.93 (0.10, 39.13)
Loss ≥20 letters	0.007 (0.000, 0.027)	0.077 (0.001,0.339)	10.47 (0.46, 237.71)

^§^Mean of 100000 samples from posterior distributions of probability for change in BCVA from baseline category.

*Ratio of mean probabilities with credible interval based on Delta method on log scale.

**Table 6:** **Bayesian method estimates of indirect probabilities of passing different visual acuity thresholds at month 1 for patients with CRVO**

Improvement or worsening at Month 1	Probability for Group ^§^ (95% CrI)		Relative risk* (95% CrI)
	Ranibizumab	Dexamethasone	
Gain ≥20 letters	0.133 (0.082,0.195)	0.063 (0.016,0.153)	0.47 (0.14,1.61)
Gain ≥10 letters and <20 letters	0.326 (0.250,0.407)	0.435 (0.247,0.638)	1.33 (0.79,2.26)
Loss <10 letters and gain <10 letters	0.511 (0.427,0.595)	0.429 (0.264,0.601)	0.84 (0.54,1.30)
Loss ≥10 letters and <20 letters	0.022 (0.005,0.053)	0.038 (0.012,0.085)	1.70 (0.37,7.84)
Loss ≥20 letters	0.007 (0.000,0.027)	0.035 (0.004,0.120)	4.70 (0.32,69.60)

^§^Mean of 100000 samples from posterior distributions of probability for change in BCVA from baseline category.

*Ratio of mean probabilities with credible interval based on Delta method on log scale.

Relative risks of dexamethasone against ranibizumab patients achieving different levels of improvement supported these results (Tables 5 and 6). The 95% credible intervals for the relative risks were wide and the results non-significant, in a classical sense. The trend was broadly in favour of ranibizumab, however.

The adequacy of the Bayesian indirect comparison was assessed by comparing the indirect probabilities for ranibizumab with the observed probabilities reported in Table 1 for BRVO and CRVO. The estimated probabilities were consistent, in magnitude and direction and within the 95% credible intervals, with the observed probabilities in all cases.

**Figure 4:**
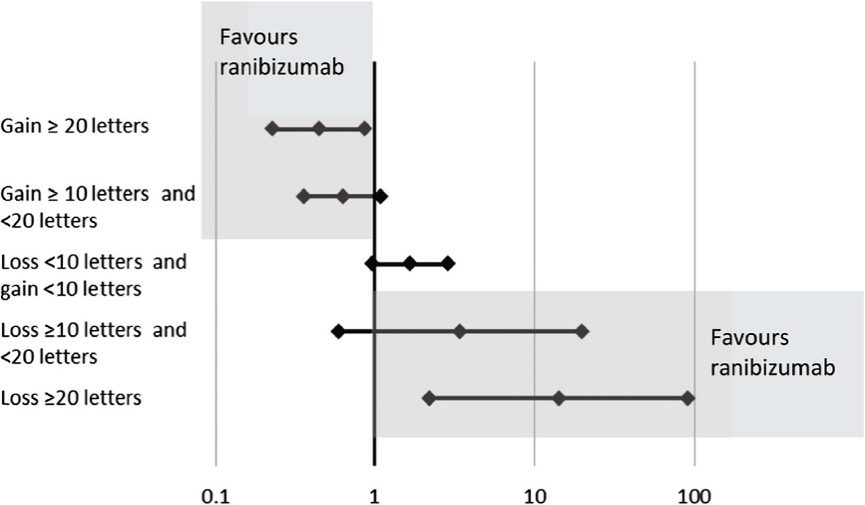
**Forest plot of indirect relative risk ±95% CI at Month 6 for patients with BRVO.** In the upper point estimates, values <1 would indicate greater chance of improving with ranibizumab; in the bottom point estimates, values >1 would indicate greater risk of worsening with dexamethasone.

**Figure 5:**
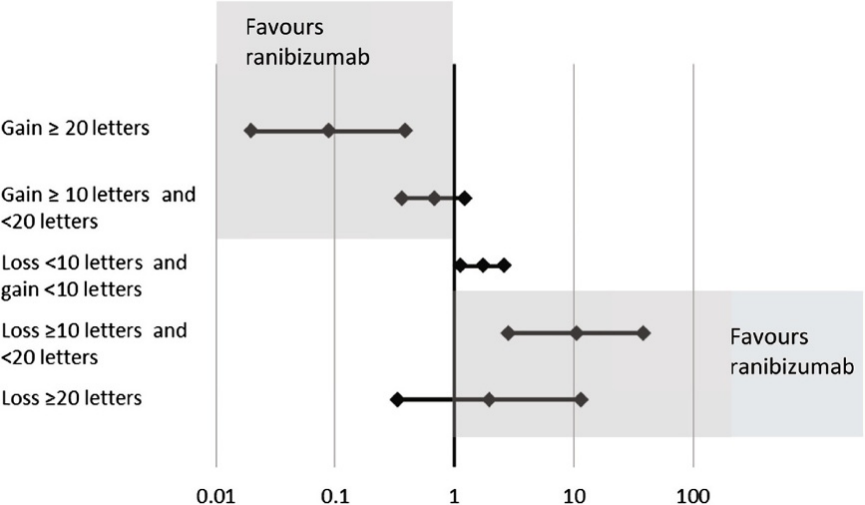
**Forest plot of indirect relative risk ±95% CI at Month 6 for patients with CRVO.** In the upper point estimates, values <1 would indicate greater chance of improving with ranibizumab; in the bottom point estimates, values >1 would indicate greater risk of worsening with dexamethasone.

The developed method was also used to compare ranibizumab against dexamethasone at 6 months, with the caveat that the option of laser treatment in the ranibizumab group of the BRAVO trial after month 3 might introduce bias in favour of ranibizumab in the BRVO setting. Results from this analysis in BRVO and CRVO are provided in Table 7 and Table 8, respectively. The comparisons continued to favour ranibizumab over dexamethasone at month 6 (Figure 4 and Figure 5).

**Table 7:** **Bayesian method estimates of indirect probabilities of passing different visual acuity thresholds at month 6 for patients with BRVO**

Improvement or worsening at Month 6	Probability for Group ^§^ (95% CrI)		Relative risk* (95% CrI)
	Ranibizumab	Dexamethasone	
Gain ≥20 letters	0.419 (0.338,0.503)	0.187 (0.086,0.316)	0.45 (0.23,0.86)
Gain ≥10 letters and <20 letters	0.353 (0.275,0.435)	0.221 (0.115,0.337)	0.63 (0.36,1.10)
Loss <10 letters and gain <10 letters	0.199 (0.136,0.269)	0.332 (0.184,0.466)	1.67 (0.97,2.89)
Loss ≥10 letters and <20 letters	0.015 (0.002,0.041)	0.051 (0.015,0.118)	3.43 (0.59,19.97)
Loss ≥20 letters	0.015 (0.002,0.041)	0.210 (0.041,0.529)	14.21 (2.21,91.25)

^§^Mean of 100000 samples from posterior distributions of probability for change in BCVA from baseline category.

*Ratio of mean probabilities with credible interval based on Delta method on log scale.

**Table 8:** **Bayesian method estimates of indirect probabilities of passing different visual acuity thresholds at month 6 for patients with CRVO**

Improvement or worsening at Month 6	Probability for Group ^§^ (95% CrI)		Relative risk (95% CrI)*
	Ranibizumab	Dexamethasone	
Gain ≥ 20 letters	0.341 (0.263,0.423)	0.031 (0.005,0.089)	0.09 (0.02,0.39)
Gain ≥ 10 letters and <20 letters	0.356 (0.277,0.438)	0.241 (0.129,0.386)	0.68 (0.37,1.23)
Loss <10 letters and gain <10 letters	0.267 (0.196,0.344)	0.463 (0.318,0.610)	1.74 (1.13,2.67)
Loss ≥10 letters and <20 letters	0.022 (0.005,0.053)	0.235 (0.111,0.402)	10.58 (2.87,38.98)
Loss ≥20 letters	0.015 (0.002,0.041)	0.029 (0.008,0.069)	1.97 (0.34,11.58)

^§^Mean of 100000 samples from posterior distributions of probability for change in BCVA from baseline category.

*Ratio of mean probabilities with credible interval based on Delta method on log scale.

## Discussion

This article describes a comparison of the efficacy of ranibizumab and dexamethasone implant in patients with macular oedema secondary to BRVO and CRVO, respectively, using a newly developed two-step model for the comparison of data from separate clinical trials. With the method, a combination of a multinomial distribution model and a Bayesian indirect comparison for multinomial outcomes, the majority of point estimates for probabilities of benefit favoured ranibizumab over dexamethasone in both BRVO and CRVO by month 1. The greater responder rates with ranibizumab were seen particularly in the segment with the biggest response. However, there were wide credible intervals due to the limited available evidence and the results were not statistically significant under a classical interpretation. The results were corroborated by consistent findings in the relative risks for receiving ranibizumab or dexamethasone, and by the analysis at month 6, although the latter may include bias in favour of ranibizumab due to possible use of laser as of month 3. A comparison at month 3 was not possible as data was unavailable. Despite these limitations, the probabilities of achieving BCVA change can be used to inform an economic model comparing ranibizumab with dexamethasone with primary outcome of change in BCVA at 1 month.

Independently of the economic model, our results are relevant for two reasons. Specifically, they provide a comparison of the efficacies of two recent treatments for macular oedema, for which no head-to-head data are available. More generally, the model may find application in other situations where outcomes from separate clinical trials are available in different formats and no direct comparison has been done.

The two-step approach was needed as the data from the clinical trials reported outcomes in different ranges, which had to be transformed to comparable scales. To achieve this, a multinomial distribution with a finer range of thresholds was fit to available dexamethasone data. This distribution was constrained to be valid so as to have probabilities summing to 1 and being non-negative. From this fitted finer distribution, the relevant probabilities could be calculated. This method for inferring a finer multinomial distribution for a set of outcomes gave an excellent fit in our dataset and has the potential to find greater use in finding compatible ranges in similar situations beyond the current analysis.

Indirect comparisons of multiple treatments via networks of evidence have become a well-developed tool for use in evidence synthesis [21]. In our dataset, the Bayesian model [22, 23] overcame several disadvantages over separately applying the standard Bucher method [19]. First, the estimated outcomes for dexamethasone and ranibizumab contained zero counts, leading to errors when calculating relative risks using the Bucher method. This is overcome by the Bayesian analysis by assigning a non-zero prior to each outcome.

Secondly, use of the relative risks within each range of change in BVCA separately to derive indirect probabilities led to probabilities >1 or summing to >1 across the categories. Although continuity corrections [24] could be used to overcome the divide by zero problem, the Bayesian model [22, 23] simultaneously models all 5 probabilities for each treatment and control, allowing them to sum up to 1 and avoiding probabilities >1 for a specific probability, thus overcoming both difficulties in a consistent manner.

As a sensitivity analysis, we performed the indirect comparison in the GENEVA setting and on the BCVA change of 5 and 15 letters categories reported in GENEVA. The results at 1 month are presented in Table 9 and Table 10 for BRVO and CRVO, respectively. For BRVO, ranibizumab had a slightly higher probability of achieving the ≥5 and ≥15 letters change outcomes. For CRVO, dexemathasone was indicated advantage on the ≥15 outcome but was roughly equivalent to ranibizumab on the ≥5 outcome. As in the base case, neither the BRVO or CRVO results were conclusive due to wide credible intervals. The results from this comparison are not strictly comparable with those performed on the 10 and 20 letters change categories as the outcomes are on different scales. As noted in the methods section, the choice of multiples of 10 letters change in BCVA was driven by the need to inform transition probabilities in a health economic model, which chose these categories as recent research has shown that decrements of this magnitude were significantly associated with changes in quality of life [12]. For this reason, the GENEVA scale was not of as much interest.

**Table 9:** **Bayesian method estimates of indirect probabilities of passing different visual acuity thresholds at month 1 for patients with BRVO on scale of GENEVA trial**

Improvement or worsening at Month 1	Probability for Group ^§^ (95% CrI)		Relative risk* (95% CrI)
	Ranibizumab	Dexamethasone	
Gain ≥15 letters	0.260 (0.140,0.414)	0.213 (0.168,0.261)	0.82 (0.45,1.47)
Gain ≥5 letters and <15 letters	0.453 (0.317,0.593)	0.470 (0.413,0.526)	1.04 (0.74,1.45)
Loss <5 letters and gain <5 letters	0.264 (0.163,0.385)	0.257 (0.209,0.308)	0.97 (0.61,1.57)
Loss ≥5 letters and <15 letters	0.018 (0.002,0.055)	0.057 (0.034,0.086)	3.27 (0.60,17.70)
Loss ≥15 letters	0.005 (0.000,0.025)	0.003 (0.000,0.012)	0.62 (0.02,18.40)

^§^Mean of 100000 samples from posterior distributions of probability for change in BCVA from baseline category.

*Ratio of mean probabilities with credible interval based on Delta method on log scale.

**Table 10:** **Bayesian method estimates of indirect probabilities of passing different visual acuity thresholds at month 1 for patients with CRVO on scale of GENEVA trial**

Improvement or worsening at Month 1	Probability for Group ^§^ (95% CrI)		Relative risk (95% CrI)*
	Ranibizumab	Dexamethasone	
Gain ≥15 letters	0.185 (0.063,0.366)	0.213 (0.149,0.283)	1.15 (0.47,2.85)
Gain ≥5 letters and <15 letters	0.434 (0.276,0.600)	0.404 (0.325,0.487)	0.93 (0.60,1.44)
Loss <5 letters and gain <5 letters	0.076 (0.037,0.135)	0.298 (0.226,0.376)	3.91 (1.92,7.98)
Loss ≥5 letters and <15 letters	0.286 (0.154,0.451)	0.043 (0.016,0.081)	0.15 (0.06,0.39)
Loss ≥15 letters	0.018 (0.002,0.061)	0.042 (0.016,0.081)	2.30 (0.33,15.85)

^§^Mean of 100000 samples from posterior distributions of probability for change in BCVA from baseline category.

*Ratio of mean probabilities with credible interval based on Delta method on log scale.

A simpler approach to conducting an indirect comparison between dexamethasone and ranibizumab would be to compare the improvement in BCVA ≥10 letters outcome which is reported by GENEVA, BRAVO and CRUISE. This is a straightforward exercise but is restricted to very little of the available evidence and is not generalizable to other settings.

As mentioned in the methods section, patients in the BRAVO trial were offered laser photocoagulation therapy at 3 months after initialisation of therapy. The indirect comparison results for BRVO at month 6 presented in Table 9 may therefore be biased in favour of ranibizumab. A comparison at 3 months would avoid this bias but data was not available to us for this time point. However, the percentage of patients achieving ≥15 letters change in BCVA has been reported for GENEVA [10], BRAVO [7], and CRUISE [8] and a simple comparison of relative risks for this outcome could be conducted. For BRVO, the relative risk for ranibizumab compared to its sham was 2.90 and dexamethasone compared to its sham was 1.61. For CRVO, the relative risk of ranibizumab compared to its sham was 4.34 and dexamethasone compared to its sham was 1.73. Uncertainties were not reported for these endpoints. This simple comparison indicates that there is evidence in favour of ranibizumab at this earlier time point but we would recommend the use of the month1 and month 6 comparisons as they are informed by a greater quantity of evidence and allow for uncertainty assessment, despite the potential bias at month 6 for BRVO.

There may be differences in expected treatment effects because of heterogeneity across populations in the different trials. The GENEVA trials had more patients with long-term macular oedema than CRUISE (72.3% <3 months) and BRAVO (67.2%) and there were slight differences in baseline visual acuities, as reported in Table 3. Other potential differences, such as severity of macular oedema and fellow eye characteristics, were not reported in all studies. These factors needed to be accounted for in the comparison to prevent biases in the analyses [25, 26]. With the Bayesian model this was achieved by generating indirect probabilities, i.e., the probabilities we would expect to observe relative to the expected response to the sham treatment if dexamethasone had been used in the ranibizumab BRAVO and CRUISE trial populations. This enables a comparison between the dexamethasone and ranibizumab outcomes. However because of the small evidence network, it was not possible to perform a Bayesian meta regression taking into account the above mentioned differences in baseline characteristics between trials.

The methodology has some weaknesses and potentials for improvement. One weakness related to using RMSE minimization and moment matching is that there are no uncertainties associated with parameter estimates so relative risks in Table 4, for example, do not have confidence intervals. Simple bootstrapping could be performed but would not be recommended due to the small size of the available data. A further disadvantage to this numerical optimization is that it is not clear whether the estimates were unbiased or efficient. The multinomial distribution could have been fitted to counts of patients achieving change in BCVA using a standard likelihood approach but this would discard data on the mean and standard deviation of the BCVA change. We chose our numerical approach to make the best use of available data but these theoretical disadvantages should be kept in mind.

An extension to our methodology, and a way to avoid the aforementioned limitations, would be a single step Bayesian approach combining observed probabilities for dexamethasone, rather than probabilities from a fitted multinomial distribution, to observed probabilities for ranibizumab. It would also be preferable to consistently use prior information in the model to constrain probabilities to be consistent. In the present work, probabilities for the control and ranibizumab groups were implicitly constrained to be consistent through their Dirichlet priors, while the dexamethasone group probabilities were explicitly scaled to sum to 1. A final problem with the method is the use of uniform non-informative priors, which are essentially adding an observation of one patient to each outcome range. This could be seen as equivalent to continuity corrections, although it also constrains probabilities to be consistent and is backed by a deeper Bayesian principle to specify a non-informative prior in the case of ignorance regarding an outcome.

A limitation specific to the current analysis was that it was based on only a very small evidence network. Only two trials were available for each indirect comparison; GENEVA and BRAVO for BRVO and GENEVA and CRUISE for CRVO. In addition to reducing the strength of the comparison, this makes it difficult to assess the impact of heterogeneity or to conduct sensitivity analyses such as omitting particular trials. It was also necessary to treat GENEVA as two separate randomised sham-controlled trials of dexamethasone in separate BRVO and CRVO populations. In the original published analysis, the GENEVA trials combined these patients into a single group and randomised them to dexamethasone or its sham as a block. The separation into two patients groups may thus overestimate the accuracy of the findings for dexamethasone as the BRVO and CRVO populations were not randomised separately.

## Conclusions

In newly developed distribution and indirect Bayesian comparison models for multinomial data from four randomised clinical trials, the majority of the point estimates favoured ranibizumab over dexamethasone in patients with macular oedema secondary to BRVO and CRVO. The differences were most marked in patients with a gain of ≥20 letters BCVA in both BRVO and CRVO. The credible intervals in all results were very wide due to the limited available data and the results were not classically significant. However, the estimated probabilities of achieving gains in BCVA at 1 month can be used to inform an economic model comparing ranibizumab with dexamethasone. Our method also provided indirect comparisons in a common clinical context in a situation where there were differences in format of reported outcomes across trials and the conventional Bucher method could not be applied. The power of this methodology recommends its use in further indirect comparisons where similar problems arise.

## Abbreviations

BCVA: Best corrected visual acuity
BRVO: Branch retinal vein occlusion
BRAVO: Ranibizuma B for the treatment of macular oedema following BRAnch Retinal Vein Occlusion: Evaluation of Efficacy and Safety
CRUISE: Ranibizumab for the Treatment of Macular Edema after Central Retinal Vein OcclUsIon Study: Evaluation of Efficacy and Safety
CRVO: Central Retinal Vein Occlusion
GENEVA: Global Evaluation of implaNtable dExamethasone in retinal Vein occlusion with macular edemA
RR: Relative risk
RVO: Retinal vein occlusion.
